# Major depressive disorder and anti-depressant therapy markedly alters the human follicular niche DNA methylome

**DOI:** 10.1530/REP-24-0467

**Published:** 2025-08-18

**Authors:** Noga Fuchs Weizman, Brandon A Wyse, Cyntia Duval, Clifford L Librach

**Affiliations:** ^1^ CReATe Fertility Centre, Toronto, Ontario, Canada; ^2^ Racine IVF Unit, Lis Maternity Hospital, Tel Aviv Sourasky Medical Center, Affiliated to Tel Aviv Faculty of Medicine, Tel Aviv University, Tel Aviv, Israel; ^3^ Department of Physiology, University of Toronto, Toronto, Ontario, Canada; ^4^ Biological Sciences, DAN Women & Babies Research Program, Sunnybrook Research Institute, Toronto, Ontario, Canada; ^5^ Department of and Gynecology, University of Toronto, Toronto, Ontario, Canada; ^6^ Institute of Medical Sciences, University of Toronto, Toronto, Ontario, Canada

**Keywords:** depression, anxiety, SSRI, DNA methylation, epigenetics, granulosa cells

## Abstract

**In brief:**

Generalized anxiety disorder, major depressive disorder and their treatment selective serotonin reuptake inhibitors (SSRIs) impact 4–17% of pregnancies worldwide and alter the epigenome of numerous tissues, but their effects on the ovarian follicle are unknown. This study profiles the methylome of granulosa cells, revealing novel epigenetic pathways and molecular mechanisms altered by mental health conditions and their treatments.

**Abstract:**

Generalized anxiety and major depressive disorders (GAD/MDD) impact 4–17% of pregnancies worldwide. GAD/MDD and SSRIs alter the epigenome of numerous tissues; however, their effect on the ovarian follicular niche is unknown. In this study, we determined SSRI concentrations in the follicular fluid and matched patients by clinical and stimulation characteristics, and then grouped them into three groups: i) treated GAD/MDD (*n* = 10), ii) untreated GAD/MDD (*n* = 4), and iii) control (*n* = 10). DNA methylation sequencing was performed on granulosa cells using the Illumina TruSeq Methyl Capture EPIC kit. For patients with untreated GAD/MDD, we identified 3,829 differentially methylated sites (DMSs). Pathway analysis revealed an enrichment in genes involved in catabolism and immune response for the hypomethylated DMSs and hypermethylated DMSs were associated with protein localization and cellular transport. When assessing the effect of SSRI treatment, we identified 3,690 DMSs. Hypomethylated DMSs were associated with genes involved in cytoskeleton organization and cellular transport, whereas hypermethylated DMSs were associated with apoptosis and cell cycle. This is the first study profiling the methylome of human granulosa cells from patients with treated or untreated GAD/MDD. This study provides a valuable dataset describing the effects of SSRI on cells in the ovarian follicular niche.

## Introduction

Generalized anxiety disorders (GAD) and major depressive disorders (MDD) impact 4–17% of pregnancies worldwide (Bennett *et al.* 2004, Ross & McLean 2006, Van de Velde *et al.* 2010, Cesta *et al.* 2016, Viuff *et al.* 2016, Dubovicky *et al.* 2017, Nemoda & Szyf 2017). Women treated for such disorders are at increased risks for negative obstetric outcomes such as miscarriages, birth defects, and preterm births (Domar *et al.* 2013, Nemoda & Szyf 2017, Evans-Hoeker *et al.* 2018). However, it is unclear whether these increased risks are driven by GAD/MDD or by their treatments (Nemoda & Szyf 2017). The correlation between GAD/MDD (Thiering *et al.* 1993, Lapane *et al.* 1995, Smeenk *et al.* 2001, Worly & Gur 2015, Akioyamen *et al.* 2016, Cesta *et al.* 2016, Evans-Hoeker *et al.* 2018, Bapayeva *et al.* 2021) and their treatments (Lapane *et al.* 1995, Friedman *et al.* 2009, Akioyamen *et al.* 2016, Casilla-Lennon *et al.* 2016, Sylvester *et al.* 2019) with fertility is yet to be established and SSRI treatment may have a positive effect on IVF success (Sylvester *et al.* 2019). Similarly, their effect on fertility treatment outcomes and the underlying mechanism for such effects have not been determined (Klock *et al.* 2004, Friedman *et al.* 2009, Sylvester *et al.* 2019).

Studies show that both pregnant people experiencing GAD/MDD and their infants have higher cortisol levels and lower dopamine/serotonin levels (Field *et al.* 2004, 2006). These led to the hypothesis that alterations to the hypothalamic–pituitary–adrenal (HPA) axis cause adverse neonatal outcomes (Liu *et al.* 2012, Doyle & Carballedo 2014). This may be driven, to some extent, by epigenetic alterations during a specific window of exposure (Devlin *et al.* 2010, Non *et al.* 2014).

Newborn behavioral syndrome, persistent pulmonary hypertension of the newborn and longer-term neurobehavioral effects have been previously linked to GAD/MDD and/or their treatments (Liu *et al.* 2012, Schroeder *et al.* 2012, Domar *et al.* 2013, Non *et al.* 2014, Viuff *et al.* 2016, Nemoda & Szyf 2017, Cardenas *et al.* 2019, Li *et al.* 2019). Most DNA methylation changes associated with maternal depression have been mapped to enhancers, pointing to regulatory functions (Nemoda & Szyf 2017). Non *et al.* identified 42 CpG sites that were mainly hypomethylated in the cord blood of neonates exposed to non-medicated MDD when compared with controls (Non *et al.* 2014, Kallak *et al.* 2021, Kallak *et al.* 2022). These hypomethylated areas were mainly associated with the regulation of transcription, translation, and cell division (Non *et al.* 2014). In one study, newborns exposed to antidepressants *in-utero* had hypomethylation in the gene body of *ZNF575*, which persisted into early but not mid-childhood. While *ZNF575* is involved in transcriptional regulation, its specific functions are largely unknown (Cardenas *et al.* 2019). Finally, a recent review found that DNA methylation modifications were associated with depression; however, the included studies varied profoundly in terms of methods of detection, tissue assayed, analysis pipelines and results; the authors concluded that more longitudinal studies using standardized experimental and laboratory methodologies were needed (Li *et al.* 2019). A critical gap in knowledge is the effects of GAD/MDD and SSRI treatment on the epigenetics of the follicular niche. The aim of this study was to evaluate the epigenetic consequences of GAD/MDD with and without the use of antidepressants on granulosa cells (GCs). These findings could serve as a proxy for understanding the potential epigenetic effects on the developing oocyte in patients undergoing IVF treatment.

## Methods

### Ethical approval

All subjects provided written informed consent for the donation of their biological waste material and collection of de-identified clinical information, including age, BMI, ovarian reserve, and treatment regimens was approved by the Veritas Independent Review Board (IRB) (Veritas #16487). The use of biological waste material for this study was approved by the Veritas IRB (Veritas #16518). All methods were performed in accordance with all relevant guidelines and regulations.

### IVF stimulation and sample collection

Patients were treated using a standard superovulation antagonist protocol. Briefly, ovarian stimulation commenced on the second or third day of the menstrual cycle with gonadotropins; gonadotropin releasing hormone (GnRH) antagonist was introduced when a lead follicle was recognized. Final ovulation was triggered by either human chorionic gonadotropin (hCG), GnRH agonist, or a combination of both. Ultrasound-guided oocyte retrieval was performed ∼36–40 h following trigger injection. Following oocyte retrieval, the cumulus–oocyte complex was isolated by an embryologist and the remaining material was processed to isolate the follicular fluid (FF) and mural GC. For this study, FF and GCs from single mature antral follicles were collected and cryopreserved individually by the CReATe Biobank (Toronto, Ontario, Canada; www.createresearchprogram.com/create-biobank/biological-materials) from consenting patients undergoing IVF–ICSI cycles at the CReATe Fertility Centre (Toronto, Ontario, Canada) between October 2020 and March 2021. Briefly, GCs were pelleted by centrifugation (700 ***g***, 10 min, 4°C). The supernatant was collected and cryopreserved as FF and used for subsequent SSRI measurement. The cell pellet was washed with a 1:1 (v:v) solution of 1X PBS and sterile water (Thermo Fisher, Canada) to gently lyse contaminating red blood cells. The washed cells were pelleted by centrifugation (700 ***g***, 10 min, 4°C) and resuspended in CryoStor CS10 freezing media (Biolife Solutions, USA) and transferred to −80°C overnight and then transferred to liquid nitrogen for long-term storage until requested by researchers for this study (Wyse *et al.* 2023).

### Measurement of selective serotonin reuptake inhibitors (SSRIs) and neurotransmitters in follicular fluid

All FF samples were assayed for SSRIs and neurotransmitters (serotonin and dopamine) using LC-MS/MS at the Analytical Facility for Bioactive Molecules (Hospital for Sick Children, Toronto, Canada). Briefly, 100 μL FF samples and standards (0–100 ng) were spiked with 100 ng internal standard, vortexed with acetonitrile and centrifuged at 20,000 *g* for 10 min at 4°C. Supernatants were transferred to siliconized conical tubes with acetonitrile and taken to dryness under nitrogen at 45°C. Dried samples were reconstituted in methanol, centrifuged as above, and cleared supernatants were transferred to glass autosampler vials and stored at 4°C until LC-MS/MS injection. LC-MS/MS was conducted using a Shimadzu UPLC system (Shimadzu, Japan) fitted with a Sciex Q-Trap 6,500 mass spectrometer (AB Sciex, USA) and used in positive electron spray ionization (ESI) mode. Samples and standards were injected on a Kinetex XB C18 column (2.6 μm, 100 Å, 50 × 3.0 mm; Phenomenex, USA). Samples were eluted using a gradient mobile phase with a total run time of 15 min. Flow rate was set to 0.4 mL/min with mobile phase A (MPA): 0.1% formic acid in water and mobile phase B (MPB): 0.1% formic acid in acetonitrile. Calibration curves (0.001–100 ng) were generated using known amounts of all compounds of interest (MilliporeSigma, USA) to permit absolute quantification. Any sample above the limit of detection (0.01 ng) was considered positive for the detected compound and assigned to the case group. Data acquisition and quantification were performed using the Analyst 1.6.2 software (SCIEX, USA).

### Inclusion criteria

Ten patients with a known diagnosis of GAD/MDD and under SSRI treatment were included in this analysis after confirming the presence of SSRIs in their FF (group 1). Four patients with a known diagnosis of GAD/MDD who were not treated at the time of sample collection were included in this analysis, after confirming the absence of SSRIs in their FF samples (group 2). Finally, the above were matched by demographic parameters (age, BMI, and ethnicity), ovarian reserve parameters (AMH and D2 FSH), and stimulation parameters (LH and E2 on trigger) with ten patients who tested negative for SSRIs and were not diagnosed with GAD/MDD (group 3). The diagnosis of GAD/MDD was obtained during routine clinical intake questionnaire before IVF treatment and patients self-reported if they were diagnosed with either GAD/MDD. Participants were Caucasian and had similar indications for infertility treatment, did not report any polysubstance abuse, all self-identified as light-to-moderate drinkers (CDC 2018), and were confirmed to be negative for cannabis consumption at the time of sample collection using liquid chromatography–mass spectrometry (LC-MS/MS) (Fuchs Weizman *et al.* 2021). Patient demographic information is presented in Table 1.

**Table 1:** Patient demographics. The means are presented ±SEM (range).

	Treated GAD/MDD (*n* = 10)	Untreated GAD/MDD (*n* = 4)	Control (*n* = 10)
Age (years)	37.4 ± 1.6 (23–42)	35.8 ± 2.4 (30–43)	37.4 ± 1.3 (30–43)
BMI (kg/m2)	25.9 ± 1.2 (18.2–29.8)	23.3 ± 0.8 (21.2–24.8)	23.6 ± 0.6 (21.1–27.6)
AMH (pmol/L)	20.2 ± 4.1 (12.0–56.8)	26.3 ± 11.9 (2.0–62.6)	25.0 ± 5.0 (7.8–59.8)
LH on trigger (IU/mL)	2.2 ± 0.5 (0.1–4.6)	3.0 ± 1.6 (0.3–8.4)	2.7 ± 0.9 (0.1–7.4)
E2 on trigger (pmol/L)	11,874.2 ± 1,134.8 (6,464–20,722)	10,513.8 ± 4,108.0 (3,008–23,685)	18,336.8 ± 3,154.9 (4,948–35,850)

### Embryological data collection

Embryological data were extracted from patients’ electronic medical records. These data included oocyte maturation, fertilization, and blastulation rates for the study participants across the three study groups (SSRI-treated GAD/MDD, untreated GAD/MDD, and controls). All data were deidentified before analysis.

### Sample preparation, DNA extraction and bisulfite sequencing

GCs from two large/dominant follicles, from the same patient, were thawed rapidly using a 37°C water bath and pooled together. Cells were washed in DMEM/F12 + 2.5% fetal bovine serum (FBS) to remove cryoprotectants. The resulting cell pellet was resuspended and used for DNA extraction using the QIAmp DNA Mini Kit (Qiagen, Germany), as previously reported (Fuchs Weizman *et al.* 2022). Samples were sequenced at the Princess Margaret Genomics Centre (Canada) using the Illumina TruSeq Methyl Capture EPIC kit (Illumina, Canada), according to the manufacturer’s instructions and as previously reported (Fuchs Weizman *et al.* 2022). Briefly, 500 ng purified genomic DNA was sheared using the Covaris S220 sonicator (Covaris, USA) to yield fragments of ∼180–220 bp. The fragmented DNA was end-repaired, poly-A tailed, ligated to unique adapters, and hybridized twice to the EPIC capture probes. Hybridized probes were purified and the libraries were bisulfite converted, amplified and further purified. Final libraries were quantified using the Qubit dsDNA HS Assay (Thermo Fisher, Canada) and quality was assessed using the 2100 Bioanalyzer High Sensitivity DNA chip (Agilent Technologies, USA). Libraries were normalized and sequenced on a NovaSeq 6000 S2 flow cell (Illumina, Canada) (paired end 2 × 100 bp).

### Bioinformatics

Differential methylation analysis was conducted utilizing the same pipeline described in Fuchs Weizman *et al.* (2022). Briefly, Trimmed reads were aligned to Human Genome Assembly 38 (hg38) using bismarck (v0.22.1) and base called using bismark_methylation_extractor (Krueger & Andrews 2011). MethylKit (v1.10.0) was used for differential methylation analysis (Akalin *et al.* 2012). Two comparisons were conducted, first the treated GAD/MDD group (group 1) vs the untreated GAD/MDD group (group 2) to elucidate the effect of SSRIs alone on DNA methylation, and second comparison was between the untreated GAD/MDD (group 2) vs the control group (group 3) to elucidate the effect of GAD/MDD on DNA methylation. CpGs were annotated to genic annotations using the annotatr package (v3.12) (Cavalcante & Sartor 2017). Differentially methylated sites (DMSs) were defined as having a percent methylation difference >25% and an adjusted *P*-value (Benjamini–Hochberg false discovery rate (FDR) method, *q*-value) <0.01. Differentially methylated regions (DMRs) were defined as regions of the genome containing at least three CpGs with a concordant (either hypo- or hypermethylated) mean methylation difference >25% between cases and controls, within a 1,000 bp interval (Watson *et al.* 2015, Fuchs Weizman *et al.* 2022). Gene set enrichment analysis (GSEA) was conducted on hypo- and hypermethylated DMSs individually for each comparison to determine the effect that DMSs have on cellular processes and functions (Subramanian *et al.* 2005). A custom gene set list created and supported by the Bader Lab (University of Toronto) was utilized, which comprised of all GO, KEGG, Reactome, and Wikipathways gene sets (v2022-10-01) (http://download.baderlab.org/EM_Genesets/) (Merico *et al.* 2010). Genes that could not be mapped to any gene set term were excluded from the analysis and gene sets with fewer than five genes and a *q*-value >0.05 were excluded from further analysis. Finally, leading edge analysis (LEA) was conducted to determine what genes were driving the enrichment scores and to highlight genes that were shared between gene sets.

### Gene expression analysis by RT-qPCR

RNA was isolated from the treated GAD/MDD group (group 1, *n* = 10), untreated GAD/MDD (group 2, *n* = 4), and the control group (group 3, *n* = 10) using the Total RNA Purification Micro Kit (Norgen Biotek, Canada). RNA was reverse transcribed using the Superscript IV VILO master mix (Thermo Fisher) and the resulting cDNA was subjected to qPCR. To assess the impact of differential DNA methylation on gene expression, eight target genes (hypermethylated regions: *CAND2*, *ATF6*, *ANKLE2*, *CDH4*, and *PGS1* and hypomethylated regions: *VDAC2*, *STMN3*, and *ASGR1*) and one reference gene (RPLP0) were selected for qPCR analysis. Pre-designed and validated PrimeTime™ qPCR assays (IDT, USA) were used for validation of NGS results. All targets were assayed in duplicate using the PrimeTime™ Gene Expression MasterMix (IDT, USA) with the cycling conditions as follows: polymerase activation at 95°C for 3 min; 45 cycles of 15 s denaturation at 95°C; and 1 min annealing/extension at 60°C. Relative fold change (ΔΔCt) was employed to quantify gene expression (Livak & Schmittgen 2001). Data analysis was performed using GraphPad Prism (version 10.3.0).

### Data analysis and literature review

We conducted a literature search of PubMed for previous studies assessing the effects of SSRIs or GAD/MDD on DNA methylation in different cell types using a variety of methods of detection and cross-referenced these to our DMSs identified within DMRs in our analysis. Genes identified in this study by pathway analysis, LEA, or previously reported to be impacted by SSRI use or GAD/MDD were further explored in depth using the Ovarian Kaleidoscope Database (Leo *et al.* 2000) and GeneCards Human Gene database (http://www.genecards.org/) to correlate our findings with hallmark processes in the ovary.

### Statistical analysis

The mean and SEM were utilized for continuous variables. Unpaired *t*-test or one-way ANOVA with multiple comparisons (Bonferroni) were used to assess the statistical significance. *P* values <0.05 were considered significant. Specific tests used are indicated in figure and table legends.

## Results

Twenty-four patients were included in this matched cohort study: ten treated cases, four untreated cases, and ten controls. QC sequencing metrics were similar between all groups (Table S1 (see the section on Supplementary materials given at the end of the article)). Demographic and clinical characteristics did not differ significantly between groups and are presented in Table 1.

FF levels of the hormonal neurotransmitters dopamine and serotonin are presented in Table S2. Dopamine was significantly lower (*P* = 0.041) in FF of patients with untreated GAD/MDD (479.5 ± 22.4 pg/mL), compared to controls (753.7 ± 67.5 pg/mL) (Table S2). Serotonin levels were significantly lower in both the treated (165.9 ± 4.0 pg/mL, *P* = 0.030) and untreated (111.0 ± 32.0 pg/mL, *P* = 0.026) GAD/MDD groups when compared to controls (432.9 ± 103.0 pg/mL) (Table S2). Notably, the FF levels of the SSRI drug escitalopram among patients with treated depression were similar to previous reports in blood (Table S2).

**Figure 1:**
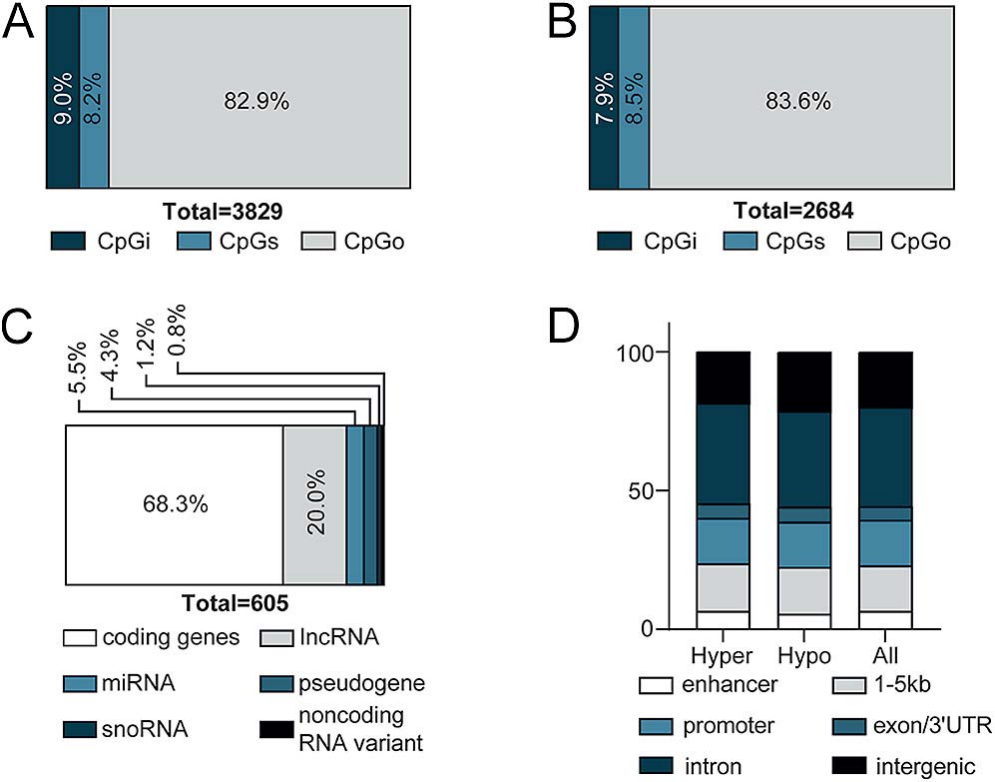
Localization and genomic feature annotation associated with DMSs in untreated GAD/MDD versus controls. (A) Of the 3,829 DMS, 343 were associated with CpG islands (CpGi) (9.0%), 313 were associated with CpG shores (CpGs) (8.2%) and 3,173 were not associated with either CpGi or CpGs and deemed CpGother (CpGo) (82.9%). (B) Of the 2,684 unique genomic features that the DMS mapped to, 212 were associated with CpGi (7.9%), 228 were associated with CpGs (8.5%) and 2,244 were not associated with either and deemed CpGo (83.6%). (C) Stratification of unique genomic feature biotypes of DMSs within ±5 kb of the transcription start site. (D) Proportions of DMS in hypo-, hyper-methylated and all DMSs associated with specific genic annotation using the ‘annotatr’ package.

**Figure 2:**
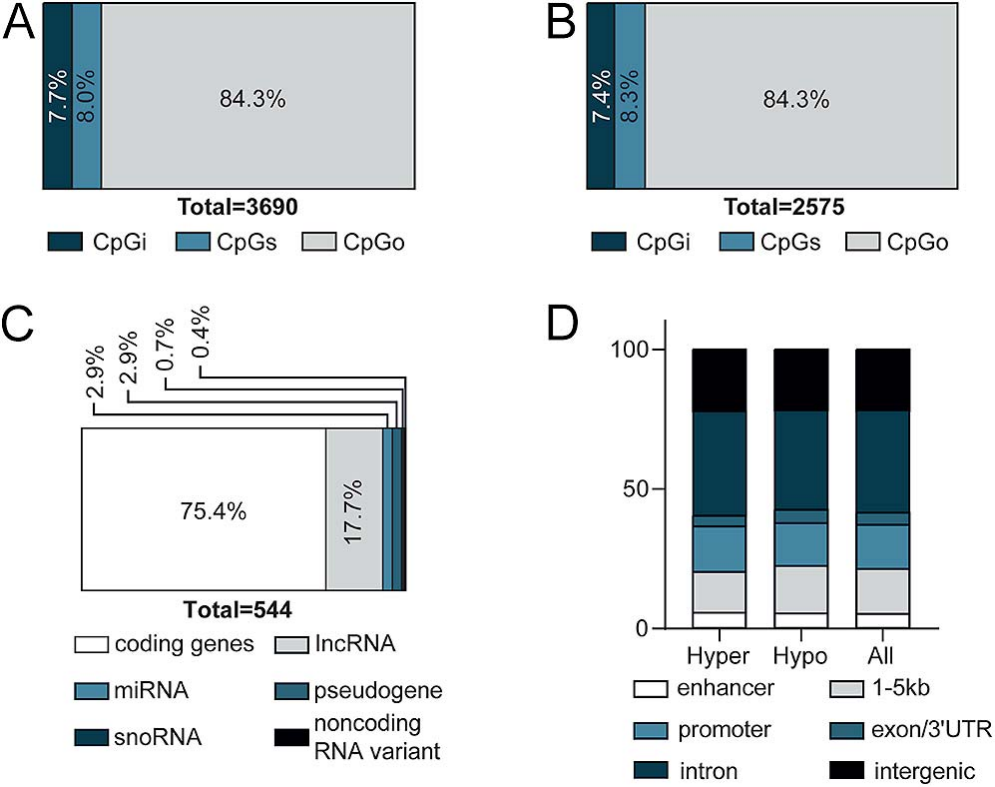
Localization and genomic feature annotation associated with DMSs in treated GAD/MDD versus untreated GAD/MDD. (A) Of the 3,690 DMS, 284 were associated with CpG islands (CpGi) (7.7%), 295 were associated with CpG shores (CpGs) (8.0%) and 3,111 were not associated with either CpGi or CpGs and deemed CpGother (CpGo) (84.3%). (B) Of the 2,575 unique genomic features that the DMS mapped to, 191 were associated with CpGi (7.4%), 214 were associated with CpGs (8.3%) and 2,170 were not associated with either and deemed CpGo (84.3%). (C) Stratification of unique genomic feature biotypes of DMSs within ±5 kb of the transcription start site. (D) Proportions of DMS in hypo-, hyper-methylated and all DMSs associated with specific genic annotation using the ‘annotatr’ package.

**Figure 3:**
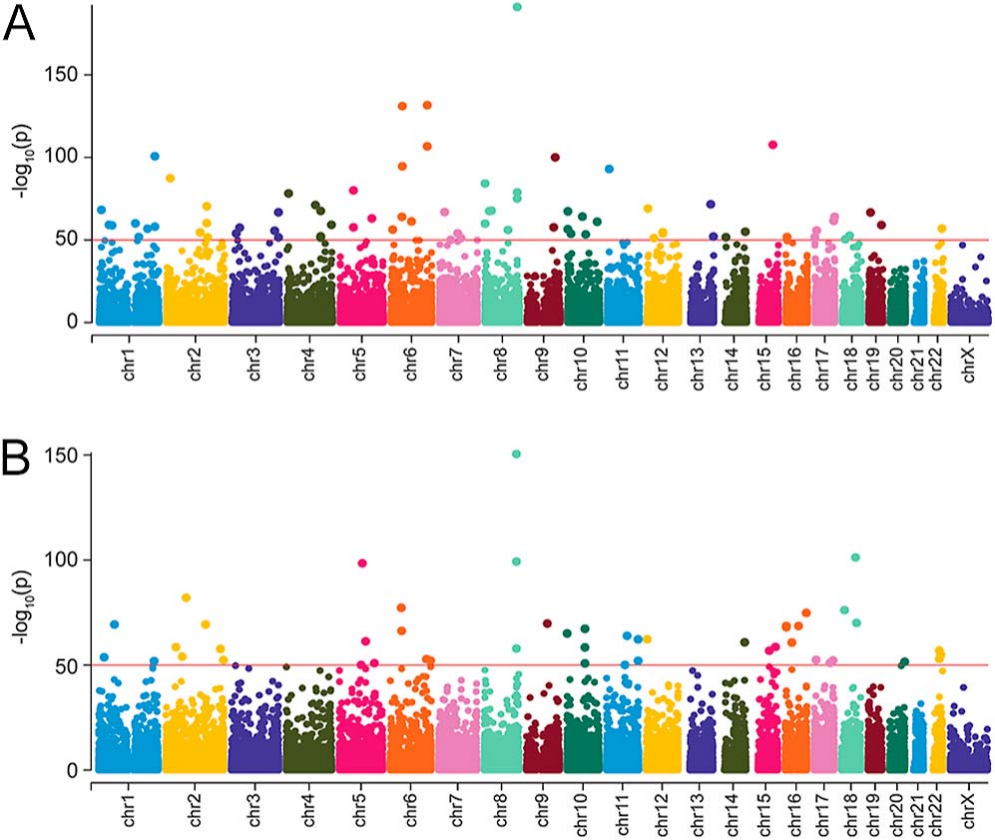
Manhattan plots of the genome-wide DMSs found in the two comparisons. All sites on these plots are significant. Significance is defined as the percent methylation difference >25% and an adjusted *P*-value (*q*-value) <0.01. The y-axis represents the −log10 (adjusted *P*). (A) Manhattan plot of all autosomes and the X chromosome in the untreated GAD/MDD versus control. (B) Manhattan plot of all autosomes and the X chromosome in the treated GAD/MDD versus untreated GAD/MDD.

Overall, when assessing the impact of GAD/MDD (untreated GAD/MDD vs control), there were 3,829 DMSs in this study, of which 1,854 were hypermethylated (48.4%) and 1975 were hypomethylated (51.6%) (Table S3). The proportion of DMS in each of the sublocations is presented in Fig. 1A and the proportion of unique genomic features, in the same sublocations, is presented in Fig. 1B. Of the DMSs within ± 5 kb of the transcription start site, two-thirds of the features were associated with coding genes (68.3%), whereas the remaining third were non-coding genes (31.7%), potentially involved in regulatory functions (Fig. 1C). There was no difference in the distribution of genic annotations between the hypo- and the hypermethylated DMSs (Fig. 1D). The same high-level overview of the DMS landscape for assessing the impact of the treatment (treated vs. untreated GAD/MDD) is presented in Fig. 2 and Table S3. Figure 3 shows a Manhattan plot depicting all CpGs that were identified for both comparisons in this study. The disease effect resulted in 70 highly significant DMSs (FDR < −log_10_50) (Fig. 3A), whereas the SSRI treatment effect resulted in 48 highly significant DMSs (FDR < −log_10_50) (Fig. 3B). No regions of the genome appeared to be highly sensitive to alterations in DNA methylation in either comparison.

**Figure 4:**
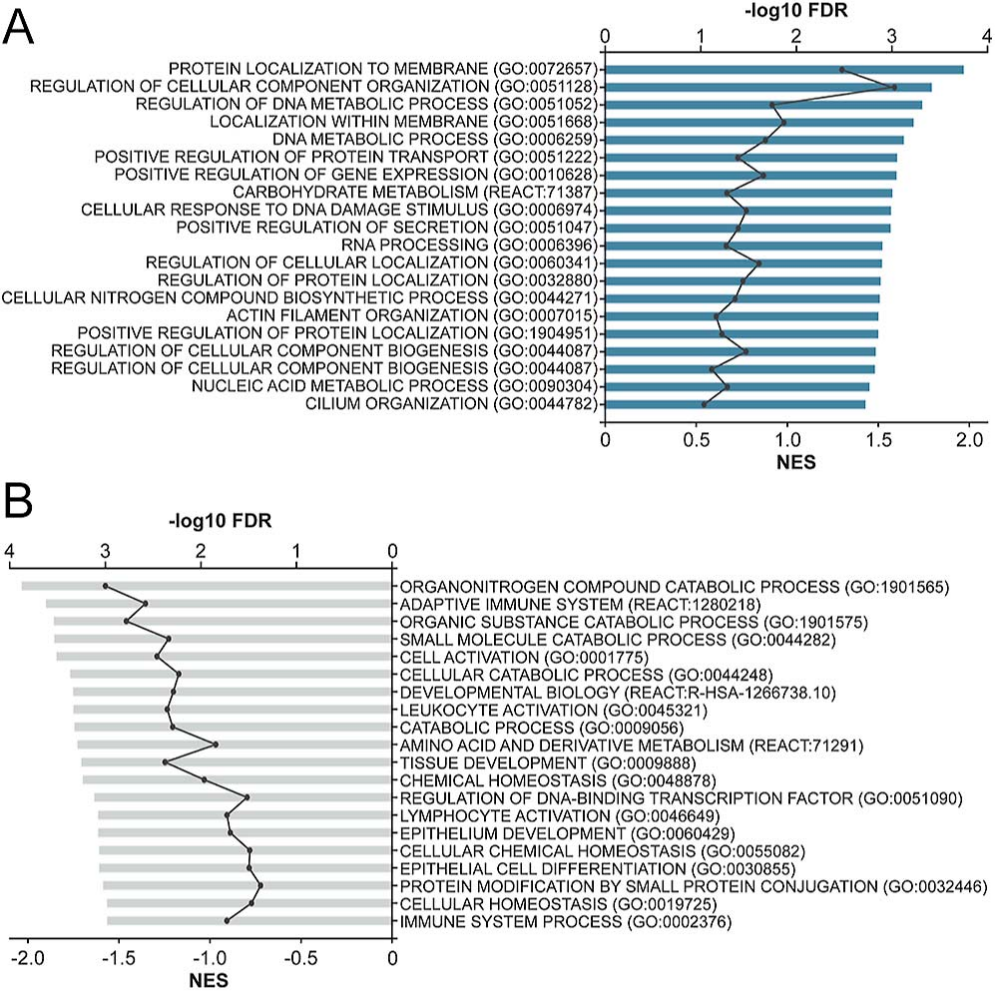
Gene set enrichment analysis of DMSs identified in the Untreated GAD/MDD versus control. Gene set enrichment analysis (GSEA) was conducted using g:Profiler-g:GOSt and interrogating KEGG, Reactome and Wikipathways databases. Genesets were considered significant if they had an adjusted *P*-value (*q*-value) <0.05 and five or more genes in the gene set. g:SCS multiple testing correction method was used to determine significance. (A) GSEA of all hypermethylated DMS. (B) GSEA of all hypomethylated DMS. A full list of gene sets can be found in Supplementary Table S5.

Next, we performed pathway analyses for both disease (untreated GAD/MDD vs control) (Table S5) and treatment effects (treated vs untreated GAD/MDD) (Table S6) using gene set enrichment analysis assessing KEGG, Reactome, and Wikipathways. Pathway analyses for disease effects (untreated GAD/MDD vs control) on hypermethylated DMSs (*n* = 1,975) showed enrichment in pathways involved in cellular and protein localization, intra- and extra-cellular transport, and response to DNA damage (Fig. 4A, Table S5), whereas hypomethylated DMSs (*n* = 1,854) showed enrichment in catabolism, immune response, and cellular homeostasis (Fig. 4B, Table S5).

**Figure 5:**
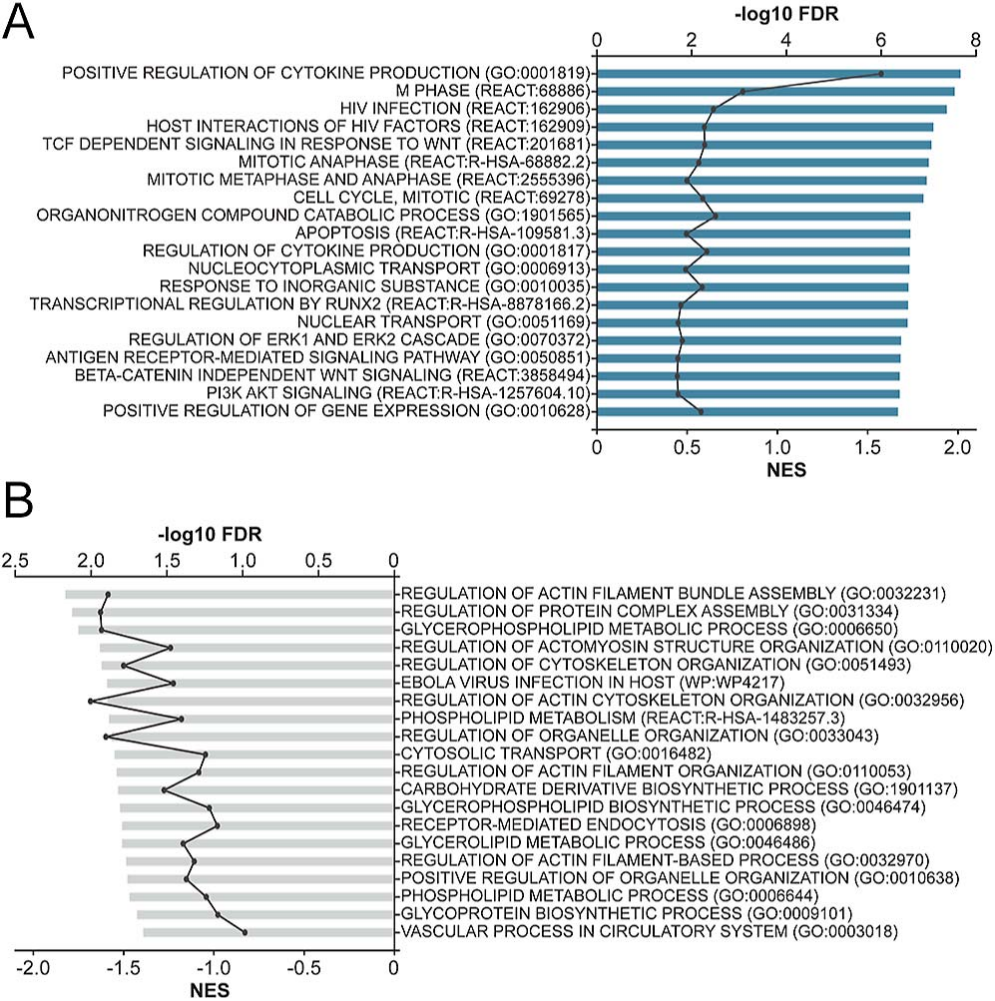
Gene set enrichment analysis of DMSs identified in the treated GAD/MDD versus untreated GAD/MDD. Gene set enrichment analysis (GSEA) was conducted using g:Profiler-g:GOSt and interrogating KEGG, Reactome and Wikipathways databases. Gene sets were considered significant if they had an adjusted *P*-value (*q*-value) <0.05 and five or more genes in the gene set. g:SCS multiple testing correction method was used to determine significance. (A) GSEA of all hypermethylated DMS. (B) GSEA of all hypomethylated DMS. A full list of gene sets can be found in Supplementary Table S6.

Treatment effect analysis of hypermethylated DMSs (*n* = 1,741) was enriched in pathways associated with apoptosis, cell cycle regulation and immune response (Fig. 5A, Table S6). Treatment effect analysis of hypomethylated DMS (*n* = 1,949) was enriched in pathways involved in cytoskeleton organization, intra- and extracellular transport, and cellular metabolism (Fig. 5B, Table S6).

To highlight loci most likely to have a biological effect, we looked at DMRs (defined as regions of the genome containing at least three CpGs with a concordant (hypo- or hypermethylated) mean methylation difference of >25% between cases and controls, within a 1,000 bp interval). When assessing the disease effect (untreated GAD/MDD vs. control), we identified 73 DMRs (Table S7), of which the top 30 are presented in Table 2. Eighteen DMRs were associated with non-coding RNAs (ncRNAs), four with transcription factors, three with apoptosis, and others involved in adipogenesis, biosynthesis, and cell signaling (Table 2). Once again, some were hypomethylated (*VDAC2, RPTOR* and *DAPL1*), while others were hypermethylated (*CAND2* and *AGPAT3*). When assessing the treatment effect (treated vs. untreated GAD/MDD), we identified 59 DMRs (Table S8), of which the top 30 are presented in Table 3. Of these, 14 are associated with ncRNAs, four with cellular metabolism, five with cell cycle and signaling, and two with reproduction (Table 3); some were hypomethylated (*STMN3* and *CD7*), while others were hypermethylated (*CSMD1*, *ANKLE2*, and *CDH4*). Notably, six DMRs were affected in different directions by the disease and its treatment, which could be the result of an insult and a correction and require further exploration (Table 4).

**Table 2:** Top 20 DMRs – untreated GAD/MDD vs control.

Location	Size (bp)	DMR state	Sig CpGs	#CpGi	#CpGs	#CpGo	*q* value	Mean MD	Max MD	Genomic feature	Main function	annotatr GA
Chr8: 134464025-134464977	952	Hyper	3	3	0	0	9.33E-192	44.73	69.83	*ZFAT-AS1*	ncRNA	Intergenic
Chr8: 134465743-134466734	991	Hypo	4	4	0	0	1.40E-79	−39.04	−49.26	*ZFAT-AS1*	ncRNA	Intron
Chr10: 75168362-75168390	28	Hypo	3	0	0	3	4.26E-54	−47.17	−53.35	*VDAC2*	Apoptosis	Promoter
Chr17: 250658-251102	444	Hypo	3	0	3	0	6.87E-49	−29.39	−31.39	*LINC02091*	ncRNA	Intron
Chr3: 12787451-12787816	365	Hyper	6	0	0	6	1.34E-44	33.01	43.81	*CAND2*	TR	Intron
Chr12: 47248949-47249295	346	Hypo	4	0	0	4	2.23E-42	−33.56	−38.62	*PCED1B-AS1*	ncRNA	Intron
Chr16: 87875035-87875071	36	Hypo	3	0	0	3	9.44E-41	−45.59	−51.09	*SLC7A5*	Transporter	Intergenic
Chr8: 54283185-54283259	74	Hyper	4	0	0	4	5.66E-37	41.68	42.39	*RNU105C*	ncRNA	Promoter
Chr4: 183013908-183013952	44	Hypo	4	0	0	4	2.54E-36	−27.70	−33.10	*CIBAR1P2*	Pseudogene	Promoter
Chr11: 67800245-67801123	878	Hypo	4	0	0	4	4.05E-35	−35.59	−39.81	*FAM86C2P*	Pseudogene	Promoter & intron
Chr2: 236855875-236856034	159	Hypo	3	0	0	3	4.17E-34	−41.56	−45.22	*LOC93463*	ncRNA	1–5 kb
Chr2: 239938929-239938957	28	Hyper	4	4	0	0	6.01E-33	34.88	42.59	*MIR4786*	miRNA	Intron
Chr4: 8556471-8556540	69	Hyper	4	0	0	4	1.12E-32	35.50	45.97	*GPR78*	CS	1–5 kb
Chr16: 88279356-88280072	716	Hypo	3	0	3	0	2.47E-32	−31.76	−35.57	*LINC02182*	ncRNA	Enhancer
Chr6: 151085999-151086136	137	Hypo	3	0	0	3	3.05E-32	−40.68	−48.90	*MIR12131*	miRNA	Intergenic
Chr21: 43961525-43961645	120	Hyper	3	0	3	0	1.45E-31	30.08	33.50	*AGPAT3*	PB	1–5 kb
Chr11: 71402909-71403089	180	Hyper	4	0	0	4	4.87E-31	30.39	34.62	*ACTE1P*	ncRNA	Intron
Chr3: 172244356-172244414	58	Hypo	3	0	0	3	9.68E-31	−31.96	−32.10	*FNDC3B*	Adipogenesis	Intron
Chr8: 3021883-3022166	283	Hypo	5	1	4	0	4.00E-30	−32.96	−37.78	*LINC03021*	ncRNA	Promoter & 1–5 kb
Chr19: 1732992-1733009	17	Hypo	4	0	4	0	6.02E-30	−47.30	−49.54	*ONECUT3*	TF	1–5 kb

Chr, chromosome; Sig, significant; MD, methylation difference; GA, genic annotation; *ZFAT-AS1*, ZFAT, antisense RNA 1; *VDAC2*, voltage-dependent anion channel 2; *CAND2*, cullin associated and neddylation dissociated 2; *SLC7A5*, solute carrier family 7 member 5; *RNU105C*, RNA, U105C small nucleolar; *CIBAR1P2*, CIBAR1 pseudogene 2; *FAM86C2P*, family with sequence similarity 86 member C2, Pseudogene; *MIR4786,* MicroRNA 4786; *GPR78*, G protein-coupled receptor 78; *MIR12131*, MicroRNA 12131; *AGPAT3*, 1-acylglycerol-3-phosphate O-acyltransferase 3; *FNDC3B*, fibronectin type III domain containing 3B; *ONECUT3*, one cut homeobox 3; TR, transcription regulation; CS, cell signaling; PB, phospholipid biosynthesis; TF, transcription factor; DMRs, differentially methylated regions.

**Table 3:** Top 20 DMRs – treated GAD/MDD vs untreated GAD/MDD.

Location	Size (bp)	DMR state	Sig CpGs	#CpGi	#CpGs	#CpGo	*q* value	Mean MD	Max MD	Genomic feature	Main function	annotatr GA
Chr8: 134465815-134466775	960	Hyper	5	5	0	0	4.97E-100	37.15	56.99	*ZFAT-AS1*	ncRNA	Intergenic
Chr10: 75168362-75168390	28	Hyper	3	0	0	3	4.96E-68	53.42	59.28	*SAMD8*	SLM	Intron
Chr6: 168229488-168229516	28	Hyper	3	3	0	0	7.50E-53	43.02	50.65	*LOC105378137*	ncRNA	Promoter
Chr20: 63645516-63645631	115	Hypo	4	0	0	4	2.60E-52	−39.77	−49.33	*STMN3*	MTA	Intron
Chr8: 3021883-3022166	283	Hyper	6	2	4	0	2.32E-48	36.14	45.95	*CSMD1*	NS/REP	Intron
Chr16: 88279356-88280072	716	Hyper	4	0	4	0	7.94E-41	42.97	47.39	*LINC02182*	ncRNA	Intron
Chr8: 134464801-134464986	185	Hyper	3	3	0	0	4.28E-40	40.75	62.61	*ZFAT-AS1*	ncRNA	Intergenic
Chr7: 36095116-36095121	5	Hyper	3	0	0	1	1.17E-39	46.27	48.76	*LOC101928618*	ncRNA	Promoter
Chr12: 132733029-132733635	606	Hyper	3	3	0	0	6.85E-38	26.62	27.29	*ANKLE2*	Cell cycle	Promoter
Chr5: 146099390-146100159	769	Hypo	3	0	0	3	2.21E-37	−35.59	−37.25	*PLAC8L1*	Keratinization	Promoter and intron
Chr4: 8556471-8556540	69	Hypo	3	0	0	3	2.24E-34	−37.50	−49.61	*GPR78*	CS	1–5 kb
Chr10: 13473094-13473335	241	Hyper	3	1	2	0	2.73E-34	36.47	41.83	*BEND7*	DNA binding	Intron
Chr8: 141252974-141253072	98	Hyper	3	3	0	0	9.02E-33	33.72	39.31	*LOC105375787*	ncRNA	1–5 kb
Chr4: 183013908-183013952	44	Hyper	5	0	0	5	3.12E-32	27.69	31.96	*CIBAR1P2*	Pseudogene	Enhancer
Chr19: 1732992-1733009	17	Hyper	4	0	4	0	9.22E-32	51.25	54.24	*ONECUT3*	TF	Intergenic
Chr19: 302255-302349	94	Hypo	3	0	0	3	1.15E-31	−35.87	−40.76	*PLPP2*	SLM	1–5 kb
Chr20 :61389938-61390813	875	Hyper	5	1	4	0	1.41E-30	34.59	39.39	*CDH4*	Cell junction	Intron
Chr3: 172244356-172244414	58	Hyper	3	0	0	3	2.17E-29	31.38	33.05	*FNDC3B*	Adipogenesis	Intron
Chr17: 82318384-82319375	991	Hypo	3	3	0	0	2.79E-29	−27.93	−32.06	*CD7*	AI	Promoter & 1–5 kb
Chr7: 158959871-158960025	154	Hyper	3	3	0	0	7.53E-29	30.79	32.37	*LINC00689*	ncRNA	1–5 kb

Chr, chromosome; Sig, significant; MD, methylation difference; GA, genic annotation; *ZFAT-AS1*, ZFAT antisense RNA 1; *SAMD8*, Sterile alpha motif domain containing 8; *STMN3*, stathmin 3; *CSMD1*, CUB and sushi multiple domains; *ANKLE2*, ankyrin repeat and LEM domain containing 2; *PLAC8L1*, PLAC8 like 1; *GPR78*, G protein-coupled receptor 78; *BEND7*, BEN domain containing 7; *CIBAR1P2*, CIBAR1 pseudogene 2; *ONECUT3*, one cut homeobox 3; *PLPP2*, phospholipid phosphatase 2; *CDH4*, cadherin 4; *FNDC3B*, fibronectin type III domain containing 3B; *CD7*, CD7 molecule; SLM, sphingolipid metabolism; NS/REP, nervous system/reproduction; MTA, microtubule assembly; CS, cell signaling; TF, transcription factor; AI, adaptive immunity; DMRs, differentially methylated regions.

**Table 4:** Common DMRs in opposing directions.

Effect/chromosome	Location	Size (bp)	DMR state	Sig CpGs	#CpGi	#CpGs	#CpGo	*q* value	Mean MD	Max MD	Genomic feature	annotatr GA	Main function
Disease effect*													
Chr3	97018895	28	Hypo	5	0	0	5	1.56E-15	−31.40	−37.29	*EPHA6*	Intron, intergenic	CS
Chr4	8556471	69	Hyper	4	0	0	4	1.12E-32	35.50	45.97	*GPR78*	1–5 kb	CS
Chr6	29680691	625	Hyper	3	0	0	3	5.38E-18	31.68	37.72	*ZFP57*	1–5 kb	TR
Chr16	87875035	36	Hypo	3	0	0	3	9.44E-41	−45.59	−51.09	*SLC7A5*	Intergenic	Transporter
Chr17	78399340	214	Hypo	3	1	2	0	9.48E-28	−45.80	−50.41	*PGS1*	Promoter, 1–5 kb	Biosynthesis
Chr19	1732992	17	Hypo	4	0	4	0	6.02E-30	−47.30	−49.54	*ONECUT3*	Intergenic	TF
Treatment effect^†^													
Chr3	97018895	28	Hyper	6	0	0	6	5.18E-13	30.62	34.70	*EPHA6*	Intron	CS
Chr4	8556471	69	Hypo	3	0	0	3	2.24E-34	−37.50	−49.61	*GPR78*	1–5 kb	CS
Chr6	29680691	625	Hypo	3	0	0	3	3.14E-15	−31.59	−35.96	*ZFP57*	Promoter, 1–5 kb	TR
Chr16	87875035	36	Hyper	3	0	0	3	4.25E-11	27.39	30.69	*SLC7A5*	Intergenic	Transporter
Chr17	78399340	214	Hyper	3	1	2	0	1.06E-16	37.79	39.31	*PGS1*	Promoter	Biosynthesis
Chr19	1732992	17	Hyper	4	0	4	0	9.22E-32	51.25	54.24	*ONECUT3*	Intergenic	TF

* Disease effect (untreated GAD/MDD vs control).

^†^ Treatment effect (treated GAD/MDD vs untreated GAD/MDD).

Sig, significant; MD, methylation difference; GA, genic annotation; CS, cell signaling; TR, transcription regulator; TF, transcription factor.

To investigate whether DNA methylation differences corresponded to transcriptional changes, we performed RT-qPCR on eight genes identified as differentially methylated. These included five genes located within hypermethylated regions (*CAND2, ATF6, ANKLE2, CDH4,* and *PGS1*) and three within the hypomethylated regions (*VDAC2, STMN3,* and *ASGR1*). The direction of gene expression changes was generally consistent with the predicted regulatory effects of DNA methylation. Genes with hypomethylated promoter regions, such as *VDAC2*, *ASGR1*, and *STMN3,* exhibited either increased expression or no change. Genes located within the hypermethylated regions, such as *CAND2*, *ATF6,* and *ANKLE2*, exhibited decreased expression (Supplementary Fig. 1).

Finally, embryological outcome data (maturation, fertilization, and blastulation rates) were collected and are presented in Supplementary Table 9. There were no statistical differences between the groups.

## Discussion

GAD and MDD are common among women of reproductive age, and so are treatments aimed at alleviating them (Bennett *et al.* 2004, Ross & McLean 2006, Van de Velde *et al.* 2010, Cesta *et al.* 2016, Viuff *et al.* 2016, Dubovicky *et al.* 2017, Nemoda & Szyf 2017). For this reason, it is crucial to gain a better understanding of the epigenetic effects of both GAD/MDD and their treatments on the follicular niche. Alterations of the epigenetic landscape of the follicle may have transgenerational impacts on the resultant offspring (Cesta *et al.* 2016, Fuchs Weizman *et al.* 2021). Early oocyte development requires the establishment of delicate methylation patterns and exposure to adverse environmental factors is likely to damage them. Gene imprinting, which is established throughout folliculogenesis, may also be disturbed if the follicular microenvironment is disordered (Dolinoy *et al.* 2007, Choudhuri *et al.* 2010, Singh & Li 2012, Xie *et al.* 2012, Rattan *et al.* 2019, Jiang *et al.* 2021). Recent human studies have shown that DNA methylation changes of hypothesis-driven candidate gene regions, such as the promoter of the serotonin transporter, are associated with maternal depression (Booij *et al.* 2015, Mendonca *et al.* 2019). Epigenome-wide association studies using blood cells show modest but significant changes in a subset of genes involved in immune function, following exposure to the above treatments; these DNA methylation changes were found mainly in enhancers, which point to regulatory gene expression effects (Kallak *et al.* 2021, 2022).

Herein, we provide the first comprehensive DNA methylation profile of human GCs from patients who are diagnosed with GAD/MDD and are treated with SSRIs and investigate how both the disease and treatment affect DNA methylation. To achieve this, we measured FF SSRI concentrations to better understand the effect that these drugs have on the immediate environment of GCs, and the resulting changes to the methylome of these cells. The SSRI drug escitalopram was detected in FF of patients with treated GAD/MDD, at similar levels to those previously reported in blood (Nord *et al.* 2013, Florio *et al.* 2017, Eichentopf *et al.* 2022). To our knowledge, our study is the first to report the presence of SSRIs in the follicular niche, and with similar levels to those reported in blood, and we conclude that the follicle is neither a privileged nor an enriched site of escitalopram, or its metabolites, and it is likely transudated along with other sera components, rather than being actively trafficked into or out of the follicle.

Furthermore, to assess the impact of SSRI treatment on dopamine and serotonin signaling in the follicle, we measured FF concentrations of these neurotransmitters. These levels are significantly lower in patients with GAD/MDD compared to controls. Serotonin is also significantly lower in patients with treated GAD/MDD, when compared with controls (Holck *et al.* 2019). Recently, increased FF serotonin levels have been associated with improved IVF outcomes, including a greater number of oocytes retrieved (Bodis *et al.* 2020). The significantly lower levels of serotonin observed in both the treated and untreated GAD/MDD groups may have a negative impact on the number of oocytes collected. However, this study was underpowered to detect such effects on embryological outcomes, and we intend on further exploring these effects in future studies.

Of the DMSs identified in this study, 52.6% were hypomethylated and 48.4% were hypermethylated in patients with untreated GAD/MDD, whereas in the treated depression comparison, 47.2% were hypomethylated and 53.8% were hypermethylated, indicating that treatment with SSRIs may induce DNA methylation globally. Interestingly, the global reduction of DNA methylation in response to GAD/MDD has been reported in leukocytes (Tseng *et al.* 2014). This study identified 3,829 DMSs associated with untreated GAD/MDD and 3,690 DMSs with treated GAD/MDD (Tables S2 and S3).

To refine our analysis, we focused on DMRs and identified 73 associated with untreated GAD/MDD, of which several regions associated with genes involved in apoptosis and cell cycle control (*DAPL1* and *VDAC2*), cellular growth (*RPTOR*), and biosynthesis (*AGPAT3, CAND2,* and *PGS1*), were identified as key processes altered by the disease. In particular, three genes of interest, *VDAC2, RPTOR,* and *DAPL1*, all code for proteins involved in apoptosis and cell cycle regulation. In the current cohort, all three were hypomethylated in patients with untreated GAD/MDD, possibly pointing to altered cell cycle regulation in this patient population. Altered apoptosis or cell cycle regulation may have severe clinical consequences on oocyte development and maturation, resulting in altered IVF outcomes, which needs to be further investigated in a follow-up study. *VDAC2* codes for a channel protein which is involved in dominant follicle development in cattle and has been previously shown to be downregulated in the hippocampus under stress in different animal models (Zielak *et al.* 2007, Glombik *et al.* 2015). *RPTOR* encodes a component of a signaling pathway that regulates cell growth in response to nutrient and insulin levels (Wang *et al.* 2022) and *DAPL1* encodes for a gene involved in cellular response to amino acid starvation and negative regulation of autophagy (Kulus *et al.* 2019, 2020).

The effect of GAD/MDD can also be seen on biosynthetic and degradation pathways in the follicular niche. Namely, *CAND2* (hypermethylated in our untreated patients) codes for a protein which catalyzes proteasomal degradation of other proteins. *AGPAT3* (also hypermethylated in our untreated patients) codes for a protein that has been implicated in the *de novo* phospholipid biosynthetic pathway.

Finally, *ATF6* (once again, hypermethylated in patients with untreated GAD/MDD) codes for an endoplasmic reticulum stress-regulated transmembrane transcription factor. Regulation of protein folding/unfolding in the endoplasmic reticulum is involved in GC activity and is critical to regulating the ovarian stress response (Takahashi *et al.* 2017). Moreover, the role of the endoplasmic reticulum has been reviewed on numerous occasions in neurodevelopmental disease, identifying a possible link between the pathophysiology of the brain and the ovary (Xiang *et al.* 2017). Finally, *ATF6* was identified as a critical gene driving the pathway analysis through LEA, further highlighting its importance in the response to untreated GAD/MDD.

Next, to explore the effect of treatment with SSRIs, we employed the same analysis strategy and identified 59 DMRs associated with treated GAD/MDD, of which regions associated with genes involved in cell cycle control (*ANKLE2* and *STMN3*), glycoprotein homeostasis (*ASGR1* and *CDH4*), and non-coding regulatory genes (*BDNF-AS* and *ZFAT-AS1*) were identified as the key processes altered by this disease.

Several regions associated with cell cycle control were identified to be impacted by SSRI treatment, including *STMN3,* which was hypomethylated in our treated patient population, and codes for a protein that has microtubule-destabilizing activity. It has been previously associated with MDD in humans (Oh *et al.* 2010). Furthermore, *ANKLE2,* a critical mitotic regulator, was hypermethylated in our treated patients, and mutations in *ANKLE2* have been previously associated with microcephaly (Elkhatib *et al.* 2017, Shaheen *et al.* 2019). Finally, *ANKLE2* has been identified as a critical gene driving the pathway analysis through LEA, underlining its potential role in the response to SSRI treatment. Again, alterations in cell cycle control may have critical consequences for the development of the follicle and oocyte.

We also observed that SSRI treatment affected metabolism glycoprotein homeostasis. *ASGR1*, which was hypomethylated in our treated population, codes for a transmembrane protein that is critical for the maintenance of glycoprotein homeostasis through endocytosis and lysosomal degradation. *CDH4*, which was hypermethylated in our treated cohort, codes for a calcium-dependent cell–cell adhesion glycoprotein which is involved in brain segmentation and organ development. Copy number variations of this gene have been associated with malformations of cortical development, thus improper methylation and expression may be critical to resulting offspring neuronal development (Wiszniewski *et al.* 2018). Finally, *CD7,* which codes for a transmembrane protein of the immunoglobulin superfamily, was also hypomethylated in our treated cohort. Changes in its levels have been previously associated with central nervous system disorders (Cacabelos *et al.* 2016). These findings should be further explored in future studies to enhance our understanding of the potential implications of GAD/MDD and treatments aimed at alleviating them on neonatal outcomes.

Of note, *CSMD1* and *BDNF-AS* were hypermethylated in our treated cohort, both of which are implicated in oocyte development and maturation (Kawamura *et al.* 2005, Jaillard *et al.* 2016, Lee *et al.* 2019). *CSMD1* expression has been previously associated with oocyte quality in mice (Lee *et al.* 2019) and with premature ovarian failure in humans (Jaillard *et al.* 2016). It is noteworthy that *CSMD1* is also highly expressed in the central nervous system and strongly associated with the dysregulation of neuropsychological responses (Schizophrenia Psychiatric Genome-Wide Association Study Consortium 2011, Zhang *et al.* 2020), and is mediated by a promoter-associated lncRNA (Steen *et al.* 2013). *BDNF-AS* regulates the expression of BDNF, which is essential for oocyte development and for neurodevelopment in humans (Kawamura *et al.* 2005) and alterations of its expression may have profound effects on oocyte development, maturation, and IVF success (Kawamura *et al.* 2005, Wang *et al.* 2011, Streiter *et al.* 2016).

Our study is limited by the probe-based enrichment method we employed as it has a preference for enriching CpGs associated with higher CpG density and may under-represent CpGs in less dense CpG regions. Furthermore, we would like to acknowledge that the study has limited generalizability due to the single-center study design and its small sample size. Hence, findings should be evaluated and explored in future larger-scale multicenter studies. Finally, this study was cross-sectional in design, and we do not know how prolonged untreated GAD/MDD or treatment with SSRIs would impact the methylome. This study is also limited by its sample size; however, by matching cases and controls according to confounding variables, we were able to minimize the inter-patient variability. This study was not designed to delineate GAD from MDD and was not powered to determine the impact of untreated GAD/MDD or SSRI treatment on IVF outcomes. A larger study, currently underway, is needed to further our understanding of the effects of GAD/MDD and SSRI treatment on the developing oocyte and on IVF outcomes. Future studies should also account for the duration of disease and treatment, focus on IVF treatment outcomes and neonatal outcomes and validate our findings using a mouse model of depression, such as chronic unpredictable stress or LPS- induced models.

## Conclusion

To our knowledge, this is the first study profiling the methylome of human GCs from patients with untreated GAD/MDD and patients treated with SSRIs. We described key genes involved in cell cycle regulation, apoptosis, metabolism, and protein folding that were affected. With the relatively high prevalence of GAD/MDD in women of reproductive age and increasing pharmaceutical treatment for these diseases, it is critical that we expand our understanding of the effects these drugs have on the developing follicle. Although our findings support an epigenetic impact of GAD/MDD and SSRI treatment on GCs, future studies are needed to confirm whether these changes affect oocyte competence and fertility outcomes. If future studies point to the heritability and functional consequences of these epigenetic modifications, it may alter the way in which we counsel and treat patients undergoing IVF treatment.

## Supplementary materials



## Declaration of interest

The authors declare that there is no conflict of interest that could be perceived as prejudicing the impartiality of the work reported.

## Funding

This work did not receive any specific grant from any funding agency in the public, commercial or not-for-profit sector. All funding was provided by the CReATe Fertility Centre through the reinvestment of clinical earnings.

## Author contribution statement

NFW and BAW designed this study. BAW performed the experiments. NFW, BAW, and CD analyzed the data and interpreted the results. NFW, BAW, and CD drafted the manuscript, with input from CLL. All authors read and approved the final version of the paper.

## Data availability

The datasets generated during and/or analyzed during the current study are available from the corresponding author on reasonable request.
