# Supplementary material for: Major depressive disorder and anti-depressant therapy markedly alters the human follicular niche DNA methylome
Source: Reproduction. 2025 Aug 18;170(3):e240467. doi: 10.1530/REP-24-0467 (PMC12365779; doi:10.1530/REP-24-0467)

Figure S1: Validation of DNA methylation-associated gene expression changes by RT-qPCR. Relative mRNA expression levels of eight genes with significant differential methylation were measured by RT-qPCR in granulosa cells. (A) Fold change of mRNA expression of differentially methylated genes identified in the Untreated GAD/MDD vs Control comparison. (B) Fold change of mRNA expression of differentially methylated genes identified in the Treated vs Untreated GAD/MDD comparison. Gene expression was normalized to *RPLP0* and calculated using the ΔΔCt method. Data are presented as mean ± SEM.

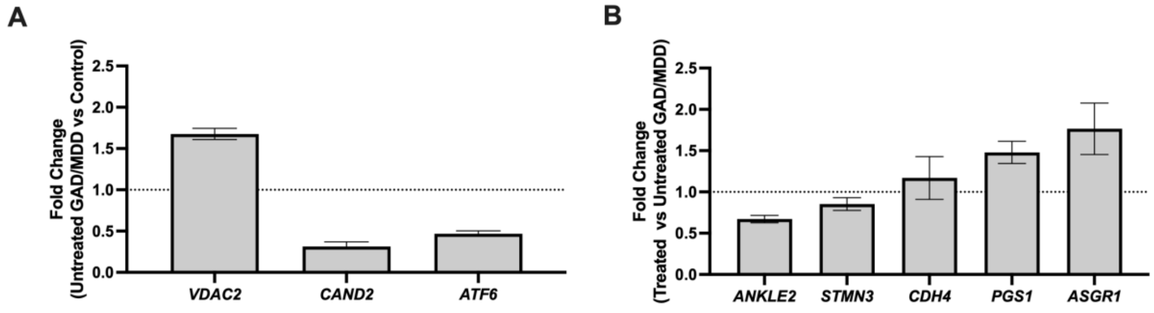

Supplement: Supplementary file 1 [file supplementary_materials.pdf]
